# Efficiency and Patient Satisfaction of Local Anesthesia Use in Surgical Circumcision in Adults—A Retrospective Cohort Study

**DOI:** 10.1155/bmri/6079986

**Published:** 2026-05-30

**Authors:** Ronen Rub, Ghalib Lidawi, Alaa Masoud, Yoav Avidor, Muhammad Majdoub

**Affiliations:** ^1^ Department of Urology, Hillel Yaffe Medical Center, Haifa, Israel, hy.health.gov.il

**Keywords:** adult patients, circumcision, local anesthesia

## Abstract

**Objectives:**

Circumcision is the most prevalent procedure in pediatric surgery, performed for therapeutic and nontherapeutic reasons. Despite extensive existing research on analgesic modalities for infant circumcision, literature regarding adult circumcision is limited and outdated. We conducted this study to examine the efficacy and patient‐reported level of satisfaction with the use of a dorsal penile nerve block (DPNB) (local anesthetic technique) for perioperative anesthesia and postoperative analgesia in an adult cohort of patients undergoing circumcision in Israel.

**Methods:**

We conducted a retrospective analysis on all cases of adult patients who underwent surgical circumcision between the years 2015 and 2020 at our institution. Data was collected with regard to efficacy and levels of satisfaction with the use of the block for circumcision in adults.

**Results:**

A total of 39 cases were included in the analysis. Our findings demonstrated perioperative efficacy of the DPNB, with 67% reporting no procedural pain. Furthermore, in spite of 12% reporting mild pain, none of the study participants required the use of general anesthesia. Postsurgery, 10% reported having no pain at all. Eighty‐seven percent of the study cohort proclaimed satisfaction with the analgesic impact from use of the local anesthesia.

**Conclusion:**

Our study findings suggest that the use of this local anesthetic technique provides adequate analgesia for surgical circumcision in adult patients, serving as a possible alternative to general anesthesia. Further research with larger study cohorts is necessary to validate our findings and evaluate whether the use of this technique improves patients′ postoperative outcomes.

## 1. Introduction

Circumcision includes the surgical removal of the penile foreskin, and it is among the most prevalent surgeries worldwide performed in young males [1–3]. The incidence varies, with up to 71% of males circumcised in the United States [4].

The surgery is performed both for therapeutic and nontherapeutic causes [2]. Primary medical indications for performing circumcisions include phimosis, paraphimosis, recurrent balanitis, and posthitis (inflammation of the prepuce) [4]. The World Health Organization (WHO) recommends circumcision as a protection from sexually transmitted diseases, such as human immunodeficiency virus (HIV), syphilis, hepatitis, herpes, and genital warts [4]. Additionally, circumcision is associated with reduced rates of penile cancer, lower incidence of urinary tract infections in infants [3], and reduced rates of cervical cancer for partners of the circumcised men [5, 6].

Nontherapeutic circumcision, typically performed for religious and cultural reasons, has been practiced for centuries, specifically in Jewish and Islamic cultures [2].

In Israel, a country populated by high rates of Jews and Muslims, statistics show that the rate of circumcision is as high as 91% of males [4]. According to Jewish law, circumcision is a ritual that must be performed for all infants on their 8th day of life unless the procedure is medically contraindicated. Neonatal jaundice is the most common reason for delaying circumcision due to medical contraindications. Other contributing factors may include low birth weight or existing infant comorbidities. These conditions often prompt a consultation with a pediatrician, who will typically provide guidance on the appropriate timing for the procedure. This ritual, according to both Jewish and Islamic traditions, dates back to Abraham and his two sons, Ishmael and Isaac. To this day, this ritual remains an integral part of both religions.

In order to minimize procedural circumcision pain, numerous anesthetic/analgesic modalities have been studied in pediatric patients, with a vast amount of research conducted on the topic [7].

Nevertheless, the literature regarding anesthesia management for circumcision in adults is very limited [8].

In this retrospective paper, we aim to examine the efficacy of local anesthesia use for surgical circumcision in an adult cohort of patients in Israel. Additionally, we hoped to assess whether the use of a local anesthetic for surgical circumcision would enhance patients′ satisfaction.

## 2. Material and Methods

We conducted a retrospective study of all adult cases of patients who underwent surgical circumcision between the years 2015 and 2020, at our institution, a university‐affiliated medical center, serving a population of half a million residents.

Ethical approval for this study was provided by the institutional review board. Institutional Review Board approval number was 0094‐21‐HYMC. As this study was retrospective in its design, informed consent and a clinical trial registry were not required.

### 2.1. Data Sources

Participants′ data were obtained from our department′s electronic and hospital medical records. Recorded characteristics included patients′ age, body mass index (BMI), surgery parameters, postoperative complications, and hospital duration.

### 2.2. Analgesic Efficacy Parameters

Data regarding the efficacy of local anesthesia use was measured with the use of the visual analogue score (VAS) obtained from the medical records during surgery and postoperatively during the patient′s admission at the surgical ward.

VAS is one of the most commonly used tools to measure pain intensity used in pain research. It has been previously validated across diverse adult patient populations, including those with chronic, acute, and perioperative pain. Its simplicity, reliability, and validity make it an optimal tool for assessing the severity level of experienced pain [9–11].

### 2.3. Patient Satisfaction Parameters

Data regarding patient satisfaction was measured with the use of a recorded satisfaction questionnaire, which was gathered from patients either during their hospital stay, during postsurgery visit, or throughout a follow‐up call. This questionnaire was conducted as part of standard routine clinical care and, as such, did not include the use of a validated questionnaire. The satisfaction questionnaire consisted of six scaled questions in a Likert structure detailing the level of analgesic satisfaction experienced during the surgery, postoperatively, and overall analgesic satisfaction. The questionnaire also included a question on the level of analgesic satisfaction in relation to the time it took to return to daily activities. An additional question is whether the patient would elect to undergo the surgery in the future under the same analgesic regimen. The last question inquired whether the patient would recommend the use of this local anesthetic treatment.

### 2.4. Dorsal Penile Block (DPB) Procedure

Our departmental protocol for local anesthesia during adult circumcision involves the use of a DPB, an effective technique for achieving regional anesthesia of the penis with a minimal local anesthetic dose [12]. All blocks included in our analysis were performed in a standardized manner by the attending surgeon, with all patients placed in the supine position with their genitalia exposed. Following prepping the skin and cleaning the gross debris, an iodine solution is applied to the penis and scrotum. With an iodine gauze pad, the shaft and the glans are cleaned in a circular motion [13]. The DPB was achieved with the use of a local anesthetic solution, injected deep into Buck′s fascia where the nerves emerge from under the pubic bone.

Following skin preparation, two injection sites were identified at the base of the penis. With the use of a 23‐G needle, a local anesthetic was injected. The anesthetic dose was determined based on patient weight, in accordance with standard safety guidelines, and adjusted for individual patient characteristics that may affect metabolism. The needle was then slightly withdrawn and redirected just below the pubic symphysis, on either side of the suspensory ligament, positioned slightly laterally and approximately 3–5 mm deeper, helping avoid the midline vessels. The syringe was then aspirated to confirm there was no flashback, ensuring the needle tip was not within an artery or vein.

Following aspiration, the attending surgeon injected the local anesthetic solution, which consisted of a mixture of 1% lidocaine (Xylocaine) and 0.5% bupivacaine (Marcaine), 10 mL at each of the sites identified in order to provide adequate analgesia for circumcision [14].

### 2.5. Anatomy of the Block

A dorsal penile nerve block (DPNB) primarily anesthetizes the dorsolateral aspects of the penis; however, the frenular region may retain sensation due to additional innervation from ventral or perineal branches of the pudendal nerve. Consequently, a dorsal nerve block alone does not always provide adequate anesthesia of the frenulum. In cases where patients experienced discomfort during the procedure, supplemental analgesia was administered using a ring nerve block to ensure adequate pain relief for the frenulum and the ventral prepuce. This additional analgesic regime was only performed selectively, in cases in which patients experienced discomfort throughout the procedure, and was not used routinely [14].

After cleaning the genital area and covering all parts of the body except for the exposed surgical area, the penile block was performed as described earlier. Anesthetic efficacy was evaluated by punching the foreskin with forceps to ensure complete analgesia coverage. The injections were generally administered laterally to allow for the circumcision of the entire penis using a local anesthetic. During the procedure, the surgeon exercised particular caution to avoid injecting too deeply or damaging any blood vessels or the urethra in order to prevent the development of a penile hematoma [12].

### 2.6. Contraindications for the DPNB

All cases of patients with a local infection at the injection site or a known allergy to local anesthetics were considered to have absolute contraindications to DPNB. Relative contraindications included bleeding disorders, anticoagulant use, and significant anatomical abnormalities; accordingly, these cases were excluded from the study cohort.

### 2.7. Surgical Procedure

The surgery was conducted using a standardized procedure. Patients were placed in a supine position. Initially, the foreskin was retracted, adhesions were removed, and smegma deposits were cleaned out. Next, the glans all the way to the preputial sulcus was disinfected with iodine prep solution. The surgeon then marked the foreskin for exterior incision in alignment with the coronal sulcus, followed by a second inner incisional mark of the prepuce 0.5 cm from the edge of the glans. Incisions were made along both markings using a 15# blade. A collar of skin was isolated between the incisions, and the skin was exposed from the underlying dartos layer. Sharp dissection, either with scissors or electrocautery, was used to separate the connections. After dissection, the shaft skin edges were sutured to the new preputial collar using absorbable sutures (Monocryl 4‐0). Finally, the area was dressed with an appropriate dressing.

An illustration of the surgical procedure involving the DPB can be found in the Supporting Information (available here).

### 2.8. Exclusion Criteria

All cases of patients who did not undergo a pain assessment or were missing a satisfaction questionnaire according to the study protocol were excluded from the analysis.

### 2.9. Statistics

Descriptive data were expressed as means and as numbers and percentages of presenting patients. SPSS and Excel (Microsoft) software were used for all statistical analyses.

## 3. Results

A total of 39 cases underwent surgical circumcision between the years 2015 and 2020 at our medical center and were included in the analysis. Patient characteristics are listed in Table 1.

**Table 1:** Demographic characteristics of the study cohort.

Parameter	Result
Age	46.5 (range 19–77)
BMI	27.9 (range 20.6–46.3)
Operation times (minutes)	48.2 (range 35–72)
Hospitalization (days)	1.2 (range 1–2 days)

Our findings showed an average surgical duration of 48.2 min (range 33–72).

The majority of our study cohort 59% chose to perform this surgery for religious and cultural reasons. Other indications for undergoing surgical circumcision included phimosis (11 patients, consisting 28% of the cohort) and skin irritations or infections (5 patients, consisting 13% of the cohort) (Table 2).

**Table 2:** Surgical indications for undergoing circumcision.

Indication for surgery	Study cohort
Religious and cultural % (no.)	59% (23)
Phimosis % (no.)	28% (11)
Skin irritations or infections % (no.)	13% (5)

None of the participants experienced any significant postoperative complications or an increased length of hospitalization, with all of the study′s cohort released from the surgical ward on the same day or the day following surgery. The mean duration of hospitalization was 1.2 days.

### 3.1. Primary Objective: Clinical Efficacy of Local Anesthesia During Surgery

Our results showed high levels of efficacy with the use of a local anesthetic (with a DPB and ring nerve block of the ventral foreskin alone) for circumcision in adult patients, with 67% (26 patients) reporting no procedural pain. Thirteen percent of the cohort (5 patients) reported feeling mild pain that did not require additional analgesia (VAS 1‐3). Twenty percent (8 patients) of the cohort reported a moderate level of pain during the operation that required additional penile block or a ring nerve block of the foreskin (VAS 4‐6). However, none of the study participants required the need of a general anesthetic.

### 3.2. Clinical Efficacy of Local Anesthesia Postsurgery

During the participants′ postoperative stay in the surgery ward, 10% (4 patients) reported having no pain at all, 79.4% (31 patients) reported experiencing mild levels of pain, and 10.2% (4 patients) reported experiencing moderate pain that required the use of analgesics (Table 3).

**Table 3:** The reported VAS score for patients performed circumcision under local anesthesia during the surgery and postsurgery.

Vas Score	0	1	2	3	4	5	6	7	8	9	10
During surgery (no. of patients)	26	2	2	1	3	2	3	—	—	—	—
Postsurgery (no. of patients)	4	8	14	9	1	1	2	—	—	—	—

*Note:* Key—VAS = 0 (no pain); VAS = 1 − 3 (mild pain); VAS = 4 − 6 (moderate pain); VAS = 7 − 9 (severe pain); and VAS = 10 (very severe pain).

### 3.3. Secondary Objective: Satisfaction Questionnaire Scores

Among our study cohort, 87% (34 patients) reported satisfaction with the analgesic impact from use of the local anesthetic technique (54% very satisfied and 33% satisfied). Ninety‐two percent (36 patients) would choose to perform the operation again under the same analgesic regimen.

All patients included in the study reported that they would recommend others to undergo surgical circumcision under local anesthesia.

The overall satisfaction levels reported by patients undergoing circumcision with the use of a local anesthetic included 82% (32 patients) reporting high satisfaction levels (55% reported very high satisfaction and 27% reported high satisfaction) and 18% (7 patients) reporting moderate satisfaction (Table 4).

**Table 4:** Patient‐reported satisfaction levels with the dorsal penile nerve block (DPNB) for pain relief during circumcision surgery and postoperatively. Additionally, it includes patient‐reported satisfaction levels for the time it took them to return to daily activities following circumcision with DPNB, alongside their overall satisfaction following circumcision with DPNB.

Level of satisfaction	Pain relief during surgery	Pain relief postoperatively	Time to return to daily activities	General satisfaction
Very satisfied % (no.)	54% (21)	46% (18)	62% (24)	56% (22)
Satisfied % (no.)	33% (13)	31% (12)	33% (13)	28% (11)
Neutral % (no.)	10% (4)	13% (5)	5% (2)	15% (6)
Unsatisfied% (no.)	3% (1)	**10%** (4)		
Very unsatisfied % (no.)	—	—		

### 3.4. Incidence of Hematoma Formation

The incidence of hematoma formation resulting from the DPB is rare. However, it can occur following inadvertent injury to the blood vessels [15]. In our cohort, we did not observe any cases of significant hematomas following the DPNB.

## 4. Discussion

This study describes the clinical efficacy and satisfaction levels with the use of a local anesthesia, a DPNB, for circumcision in an adult Israeli cohort. Our main study findings indicated that the use of a DPNB as the sole anesthetic modality provided a satisfactory analgesic effect for surgical circumcision in adults, measured by participants′ reported satisfaction levels.

Despite the frequency of circumcision, significant variability exists in its analgesic management [14, 15]. To date, circumcision has been carried out with the use of general and local anesthesia in infants and pediatric patients. Local anesthesia has been shown to be an equally effective technique, with reduced complications, compared with general anesthesia [7, 15–17]. Since it was first described in 1978 by Kirya and Werthmann, the DPNB has become a favored local anesthetic method providing perioperative and prolonged postoperative analgesia for infant circumcision [18–20]. The technique is applied by injecting a local anesthetic subcutaneously in the midline just below the symphysis pubis and proximal to the root of the penis or laterally on both sides at the base of the penis [18]. Typically, the block is administered by the attending surgeon; however, the method requires skilled expertise, and therefore, the efficacy of the block can vary. The failure rate associated with the use of a DPNB is 4%–8% [18, 21, 22].

To date, publications regarding the anesthetic management for surgical circumcision in adult patients have been limited and outdated.

In the year 1994, Serour et al. conducted a prospective randomized trial in 250 adult patients undergoing circumcision, in which they examined the use of a DPNB of the penis with and without the use of the ventral block. Eight participants required supplemental analgesia, representing a 3.2% failure rate. Similar to our findings, the authors reported that the majority of patients experienced high satisfaction with the DPNB, without the need for additional supplemental analgesia, in conjunction with low complication rates [23].

In the year 1994, Szmuk et al. evaluated various techniques of the DPNB to determine which was most effective for circumcision in a prospective randomized study in 250 adult patients. Szmuk et al. concluded that a subcutaneous ring block and a dorsal nerve block, with infiltration of the frenulum, were among the most effective local anesthesia techniques for circumcision [8, 24, 25].

In line with our results, Holman and Stuessi, in a review paper published in the year 1999, indicated that a DPB (or subcutaneous ring block) provides an adequate anesthetic effect for circumcision for an adult patient [14]. In our paper, we provided the DPNB with additional supplemental analgesia in a selective handful of cases in which patients experienced discomfort during the procedure. The DPNB primarily targets the dorsolateral aspects of the penis. The dorsal penile nerve is included in the pudendal canal alongside the pudendal artery. Within the canal, the pudendal nerve divides into its terminal branches, including the dorsal penile nerves and the perineal branch. The penile innervation originates from Sacral Nerve Roots S2–S4 via the pudendal nerve, which courses through the pudendal canal alongside the pudendal artery. The dorsal nerve on each side passes beneath the inferior ramus of the pubis, deep to the suspensory ligament, within a distinct fascial compartment that rarely communicates with its contralateral counterpart. It then continues distally within Buck′s fascia, coursing alongside the dorsal penile vessels. The frenulum of the penis, in addition to receiving sensory input from the dorsal penile nerves, also receives innervation from a branch of the perineal nerve. As such, the DPNB does not always provide sufficient analgesia [14].

The failure rate of DPNB is reported to range from 5% to 15% [26]. Failure may occur if the injection is too superficial, inaccurately placed, insufficient in volume, or affected by anatomical variation. In our cohort, we did not observe any failure rates, as none of the cases required conversion to general anesthesia; however, there were a few cases with mild discomfort. These cases were possibly due to the innervation from a branch of the perineal nerve.

More recently, in the year 2009, Long et al., in a prospective study, examined the sensory innervation of the penis using sequential blocking of the dorsal and ventral nerve components in 13 adults undergoing circumcision. Long et al. recommended the use of a DPNB in combination with a ventral infiltration of local anesthetic at the site of incision, in order to ensure sufficient analgesia of the foreskin [18].

In a retrospective analysis by Schnabl et al. on 95 patients (both children and adults), between the years 2005 and 2010, the safety of a penile block using a local anesthetic with epinephrine was evaluated. The authors reported the use of penile ring block with subcutaneous infusion anesthesia (consisting of ropivacaine and lidocaine) plus epinephrine to be a safe and effective analgesic method for circumcision. Contrary to the general view, the authors demonstrated no associated risk of necrosis related to using a subcutaneous penile ring block with epinephrine. Like our findings, the authors also showed high levels of patient satisfaction and low complication rates [25].

These reports mentioned above further elaborate on our paper′s findings, providing recommendations on the optimal modality in which to perform the DPNB, in order to achieve an adequate analgesic effect.

In contrast to our report, a prospective randomized trial, conducted in 2011 by Malkoc et al., involving 89 adult patients undergoing circumcision, examined three different techniques of local anesthesia: firstly, circular infiltration; secondly, circular plus dorsal penile infiltration; and finally, circular plus ventral upward infiltration. Their results demonstrated the usefulness of the circular plus ventral upward anesthesia technique for local anesthesia in circumcision [19].

Furthermore, this study provides unique insight into participants′ preferences and satisfaction levels with the use of DPNB for postcircumcision analgesia in adult patients. Our analysis suggested that the use of a DPNB provides sufficient postoperative analgesia, with only 15% reporting mild postoperative pain. To the best of our knowledge, these findings were not previously examined, and our study is the first to report such findings.

In a recent 2025 retrospective study, Wang et al. reported on a novel circumcision technique for adult phimosis that integrates the advantages of three existing methods—dorsal slit, guillotine, and sleeve (DGS). The authors demonstrated reduced complication rates and improved cosmetic outcomes [27].

While hematoma formation is uncommon after DPNB, it can occur if the dorsal penile vessels are inadvertently injured. The reported incidence of hematoma formation and minor bleeding varies from 1% to 5%. A recent meta‐analysis estimated an overall hematoma risk of approximately 5%, whereas earlier pediatric circumcision studies reported only minor, self‐limited, and clinically insignificant bleeding or hematoma [26, 28]. In our cohort, we did not observe any cases of significant hematomas. To minimize this risk, we implement several precautionary measures, including aspirating before injection, using a fine‐gauge needle, limiting the number of punctures, and applying compression immediately afterward.

Our paper reflects unique insights, being one of the few to explore satisfaction with an analgesic modality for circumcision in an adult population from a Middle Eastern country with a significant Muslim and Jewish population. Hence, our findings may be influenced by specific geographic and demographic factors in the region.

Of note, an interesting study finding was the high percentage of patients (59%) electing to undergo the surgery for religious and cultural reasons. A possible explanation may be the high level of uncircumcised Russian immigrants [29] existing in our cohort, who may have felt a social pressure to circumcise in order to help them integrate into Israeli culture.

This finding may demonstrate cultural health considerations, as many of these patients might not have been religiously observant at the time of circumcision. Moreover, despite our study being based on a small sample and lacking a control group, it highlights the clinical relevance of this analgesic modality in contemporary practice. Limitations of the study include its nonrandomized retrospective nature and the small cohort of patients included in its analysis. Moreover, due to the retrospective design of this study, we were limited to the use of a nonvalidated questionnaire to assess patient satisfaction.

Despite the study′s small cohort, this manuscript demonstrates a simple technique associated with high levels of patient satisfaction, with low complication rates, and provides comparable anesthetic outcomes without the need for general anesthesia. This study adds value to the limited and outdated literature on perioperative and postoperative pain management practices for surgical circumcision in an adult cohort. Our findings elaborate on the currently limited analgesic data available to primary care physicians who may encounter adults or infants either scheduled for or having undergone circumcision.

In conclusion, the use of a DPNB for local anesthesia may provide adequate and prolonged analgesia for surgical circumcision in adult patients and may be a suitable alternative to general anesthesia. Further randomized controlled trials with larger sample sizes are necessary to validate our study findings and to evaluate whether this technique can improve patients′ postoperative outcomes.

## Author Contributions

All authors help with data analysis, manuscript writing, and editing.

## Funding

No funding was received for this manuscript.

## Conflicts of Interest

The authors declare no conflicts of interest.

## Supporting information


**Supporting Information** Additional supporting information can be found online in the Supporting Information section. Step‐by‐step photographs illustrating the surgical procedure.

## Data Availability

The data that support the findings of this study are available on request from the corresponding author. The data are not publicly available due to privacy or ethical restrictions.
