# Supplementary material for: Efficiency and Patient Satisfaction of Local Anesthesia Use in Surgical Circumcision in Adults—A Retrospective Cohort Study
Source: Biomed Res Int. 2026 May 30;2026:6079986. doi: 10.1155/bmri/6079986 (PMC13239028; doi:10.1155/bmri/6079986)
Supplement: Supplementary file 1 — Supporting Information Additional supporting information can be found online in the Supporting Information section. Step‐by‐step photographs illustrating the surgical procedure. [file BMRI-2026-6079986-s001.docx]

**Appendix 1: Pictures detailing surgical technique**


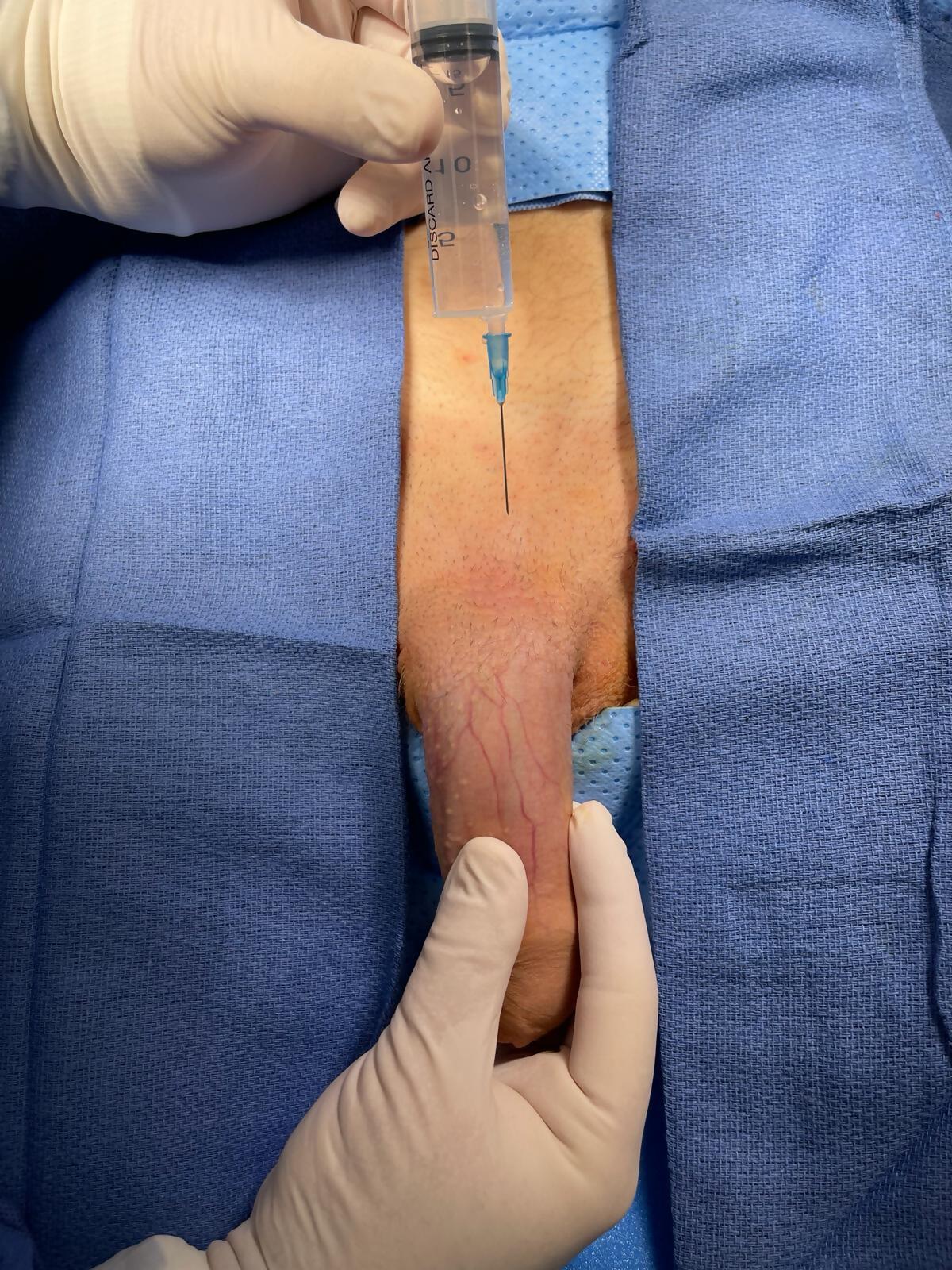


Injecting Local anesthesia at the base of the penis


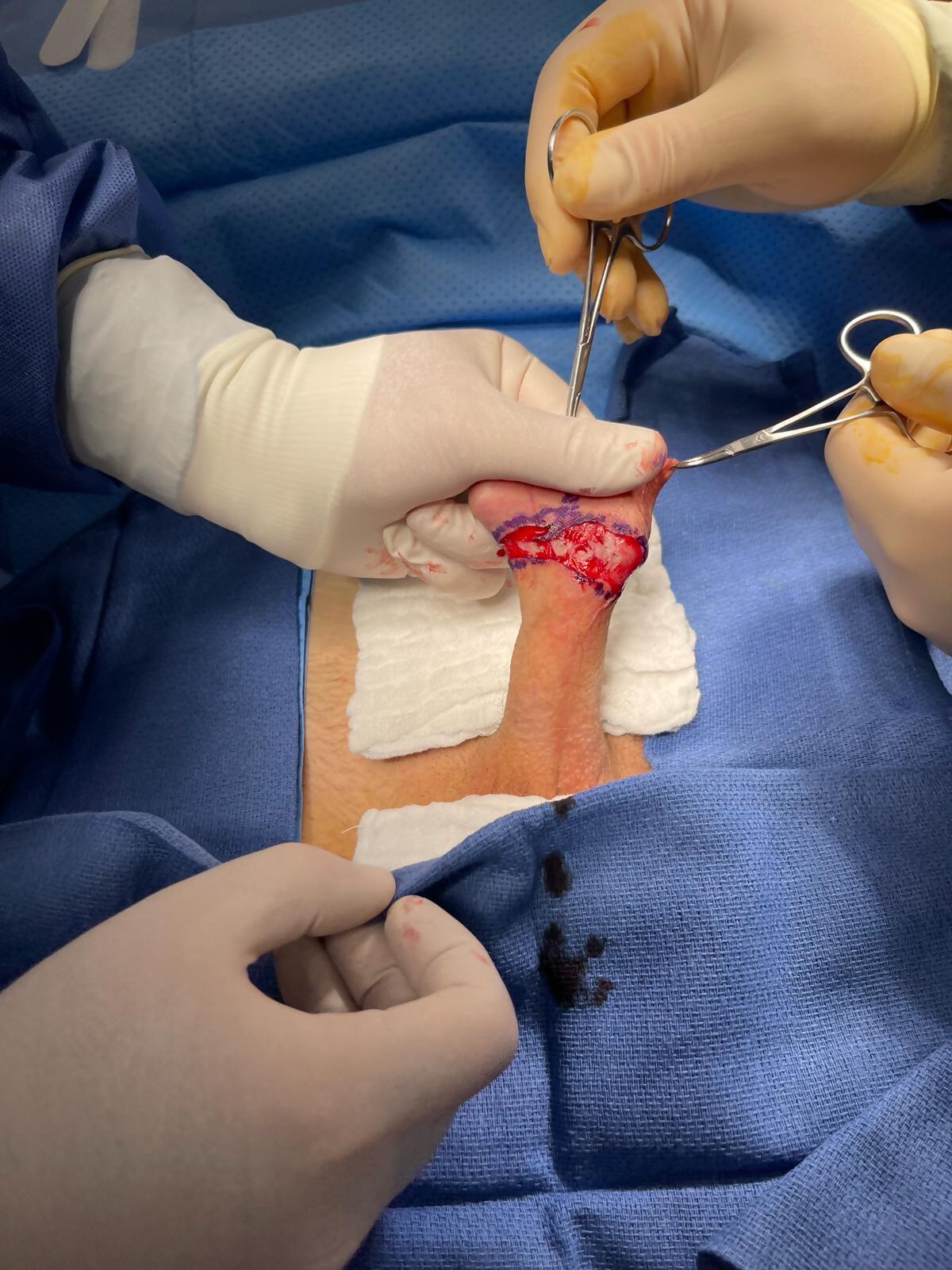


After marking the exterior foreskin over the corona of the glans penis and marking the prepuce 0.5 cm from the coronal sulcus, 2 circular incisions were made over the marked sites.


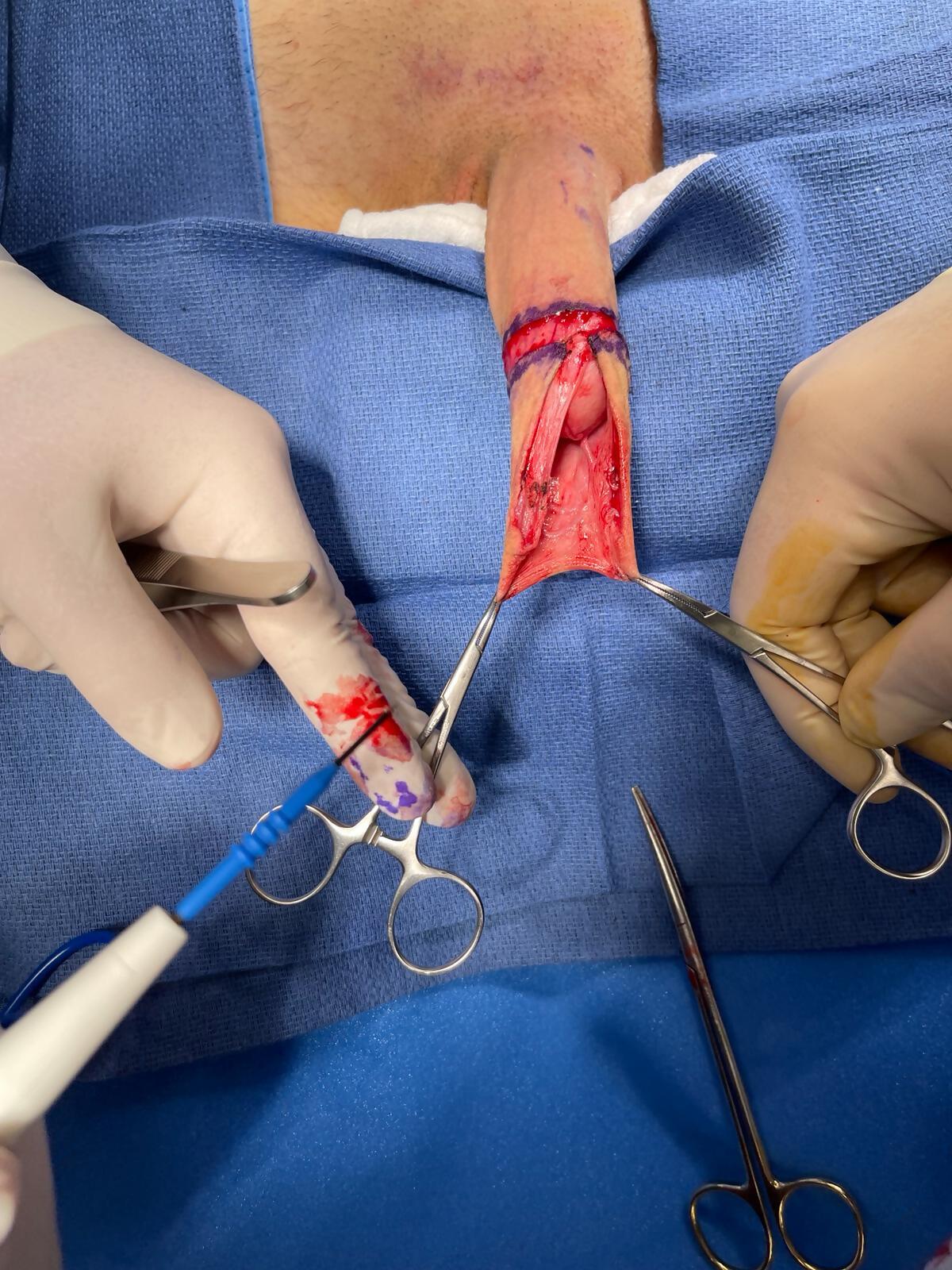


Dorsal slit using scissors or electrocautery to connect the exterior and interior incisions.


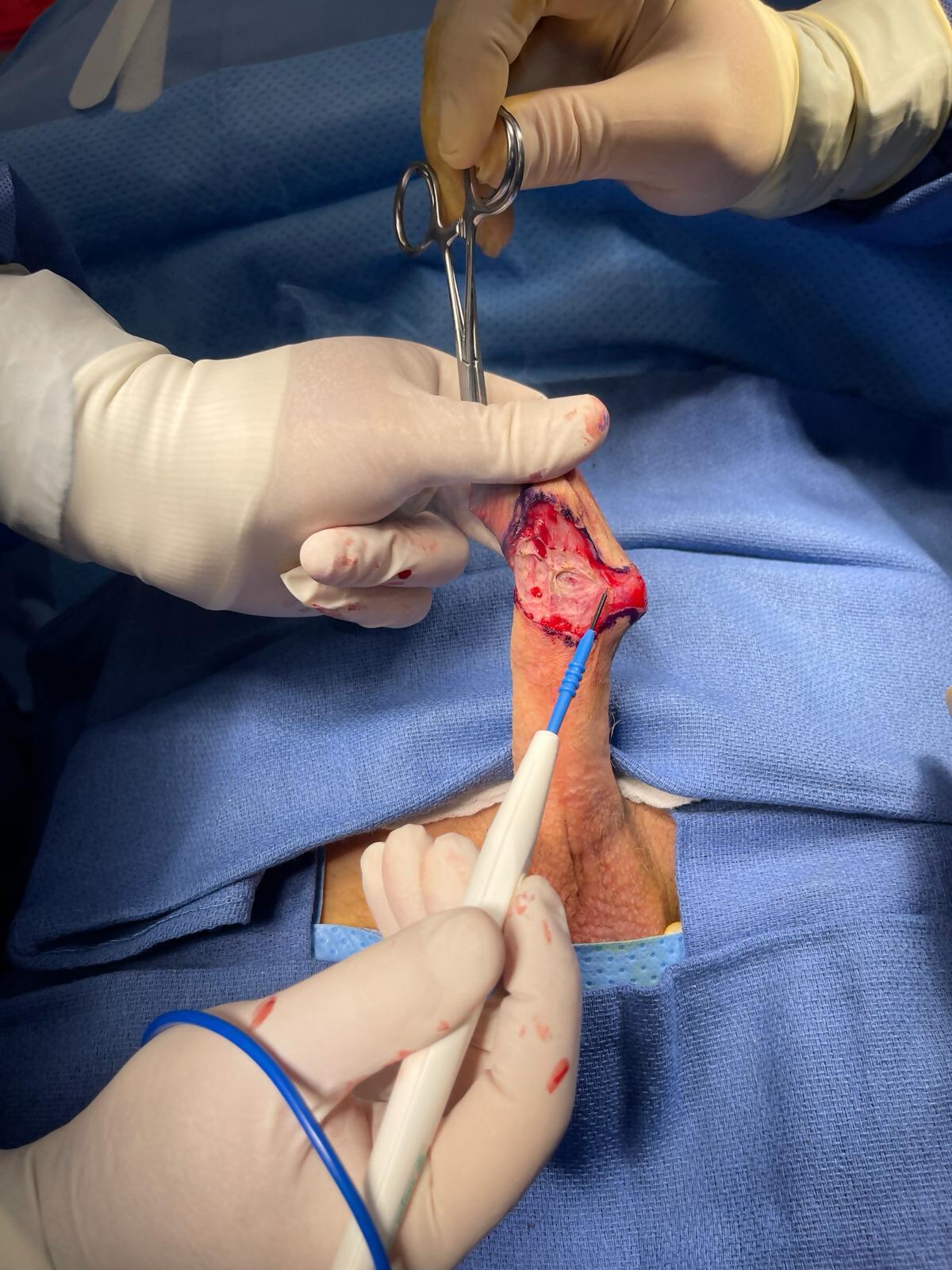


Removing the foreskin between the marked incisions using the electrocautery


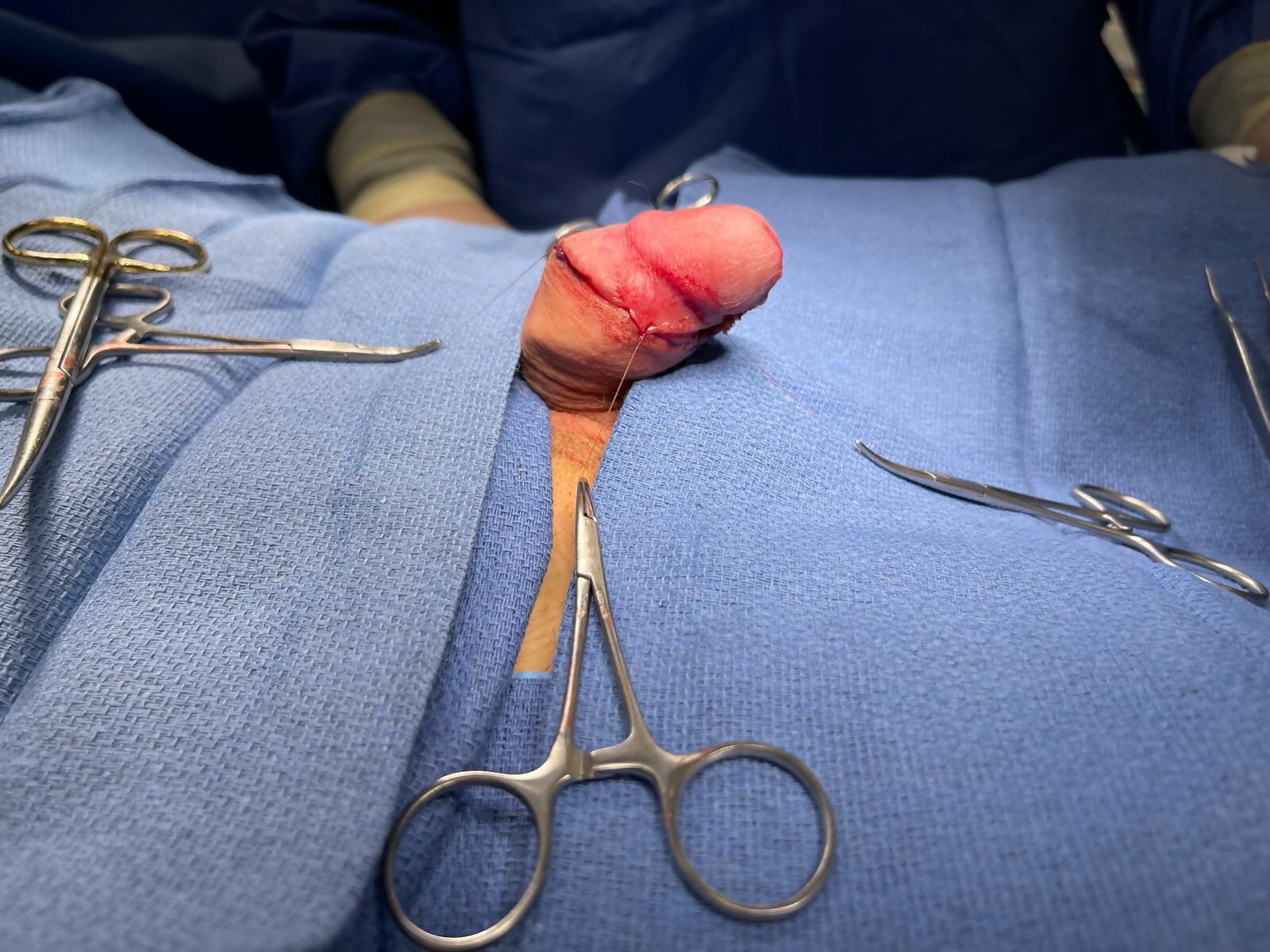


Skin edges were approximated with absorbable sutures.


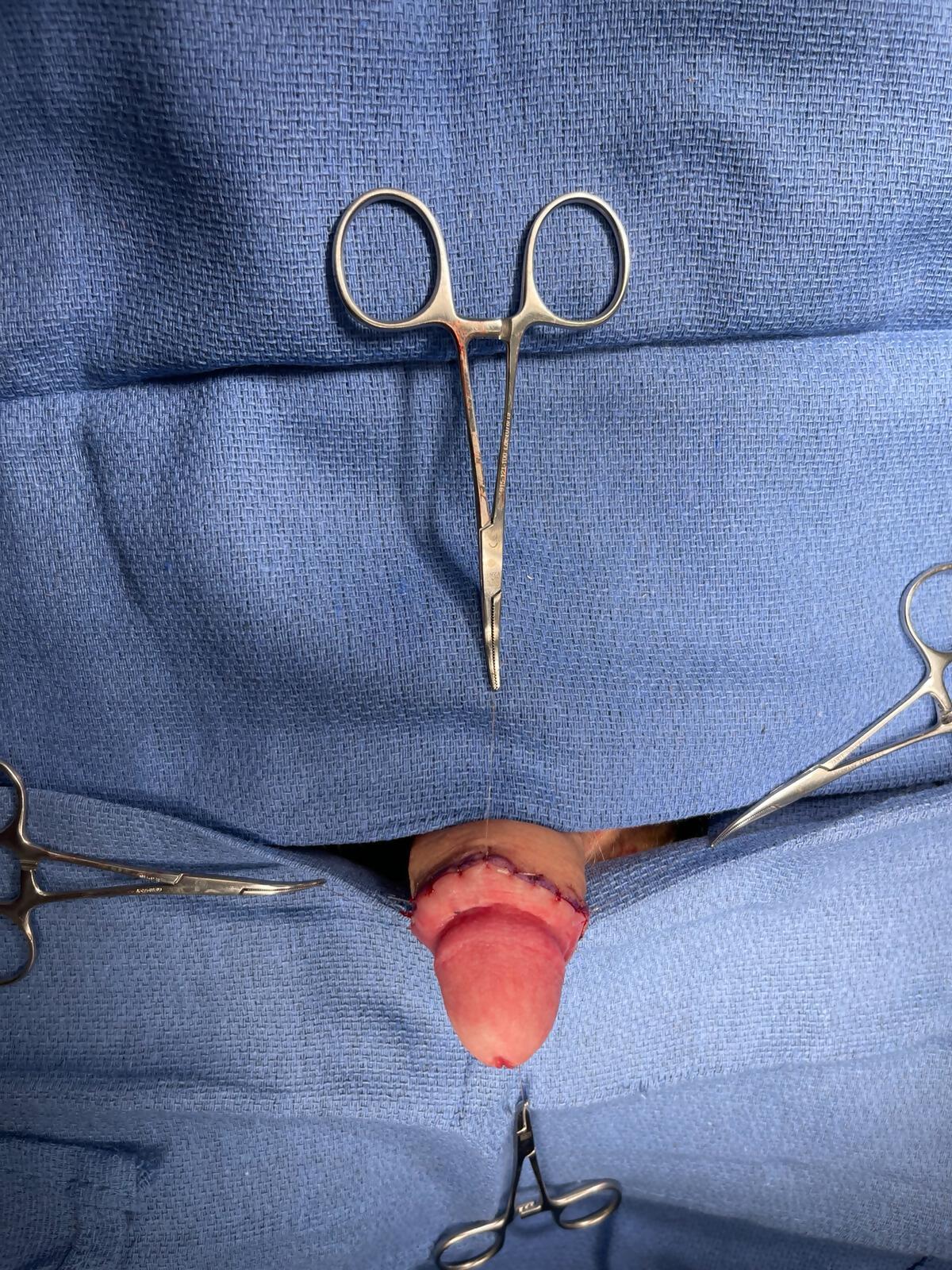


Circumscised penis .
